# The effect of cold priming on the fitness of *Arabidopsis thaliana* accessions under natural and controlled conditions

**DOI:** 10.1038/srep44055

**Published:** 2017-03-09

**Authors:** Jelena Cvetkovic, Klaus Müller, Margarete Baier

**Affiliations:** 1 Dahlem Center of Plant Sciences, Plant Physiology, Freie Universität Berlin, 14195 Berlin, Germany; 2 Meterology, Freie Universität Berlin, 12165 Berlin, Germany

## Abstract

Priming improves an organism's performance upon a future stress. To test whether cold priming supports protection in spring and how it is affected by cold acclimation, we compared seven Arabidopsis accessions with different cold acclimation potentials in the field and in the greenhouse for growth, photosynthetic performance and reproductive fitness in March and May after a 14 day long cold-pretreatment at 4 °C. In the plants transferred to the field in May, the effect of the cold pretreatment on the seed yield correlated with the cold acclimation potential of the accessions. In the March transferred plants, the reproductive fitness was most supported by the cold pretreatment in the accessions with the weakest cold acclimation potential. The fitness effect was linked to long-term effects of the cold pretreatment on photosystem II activity stabilization and leaf blade expansion. The study demonstrated that cold priming stronger impacts on plant fitness than cold acclimation in spring in accessions with intermediate and low cold acclimation potential.

*Arabidopsis thaliana* has spread over the northern hemisphere in post-glacial times and colonized habitats from cold continental areas to warm maritime ones^1^,^2^. With separation and adaptation, the radiating populations partly lost or modified their cold tolerance mechanisms in warmer areas, while they maintained and specified them in cold ones^3^,^4^,^5^.

Arabidopsis activates cold acclimation in response to persisting or oscillating cold stress^6^,^7^. It is quickly lost, when the temperatures increase^8^,^9^. As shown recently^10^, Arabidopsis can nevertheless memorize an earlier (priming) cold stress for several days over a stress-free period.

Plants sense cold e.g. by changes in the membrane fluidity, by membrane proteins, photosynthetic imbalances and changes in metabolic activity^11^,^12^,^13^,^14^,^15^. Subsequently, Ca^2+^, cAMP, hormone and ROS signaling and the CBF (*C-REPEAT BINDING FACTOR*)-regulon are activated^15^,^16^,^17^,^18^,^19^,^20^. Finally, osmolytes and stress protection proteins accumulate and the concentration of unsaturated fatty acids increases^21^,^22^. These responses protect the plants against chilling stress and often harden them also against subzero temperatures (freezing stress)^6^,^23^.

The speed and extent of cold acclimation and deacclimation vary between Arabidopsis accessions^4^,^8^,^24^,^25^,^26^. Accessions with fast and strong activation of cold acclimation also deacclimate more slowly^8^. Expression of genes encoding enzymes of the plastid antioxidant system (PAS) is either more decreased or poorly induced by cold in these accessions,. In response to weaker antioxidant protection, more reactive oxygen species (ROS) accumulate and the ratio of free radicals to H_2_O_2_ shifts stronger upon deacclimation^24^. Post-cold regulation of plastid ascorbate peroxidases mitigates chloroplast-to-nucleus ROS signaling and limits cold induction of target genes for ROS signaling for several days^10^. We hypothesized that cold priming of chloroplast-to-nucleus signaling is especially important in spring. In the first weeks of the new vegetation period, the temperature and the light intensity increase. The environmental parameters strongly fluctuate and differentially influence the signaling hubs, which regulate plant development^27^.

To evaluate the costs and benefits of a cold pretreatment in a natural environment, we compared cold (4 °C) pretreated and naïve plants of seven *Arabidopsis thaliana* accessions with different cold acclimation potentials in the field in four experimental series. Two started in the first days of March and two in the begin of May in two subsequent years. For control, plant sets of the same size and composition were cultivated in a temperature and humidity controlled greenhouse. Three of the seven accessions, namely N13, Ms-0 and Kas-1, strongly activate CBF-regulon controlled cold acclimation^8^,^28^. The other four accessions, WS, Col-0, Van-0 and Cvi-0, have an intermediate to low cold acclimation potential^8^,^28^ and express and regulate PAS genes stronger in the cold^24^,^29^. Plant robustness and reproductive fitness (seed production per plant) were determined by analysis of photosystem II activity, growth parameters, induction of flowering and seed yields.

## Results

Seven *Arabidopsis thaliana* accessions with gradually different cold acclimation potential were transferred to the field in the traditional Dahlem agricultural research area and to a close-by greenhouse after a 2 week long cold pretreatment at 4 °C. The plants were grown in randomized patterns with not cold-treated (naïve) 4 week old plants, which were indistinguishable from cold-treated ones in leaf number, weight and pigmentation^10^.

### Weather conditions

**Figure 1:**
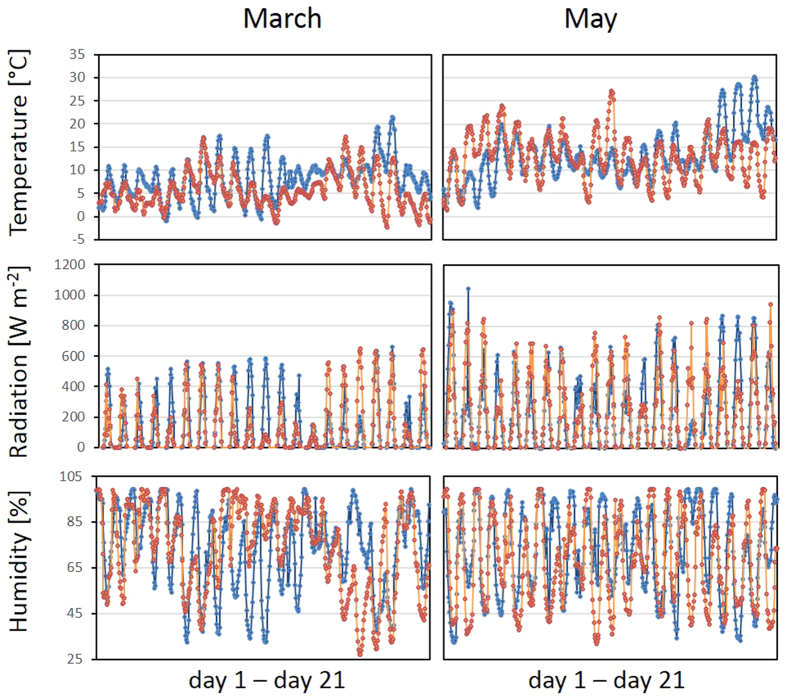
Weather data for the first 3 weeks in March and May (selected from Suppl . 1). The mean temperature (top), global radiation (middle) and relative humidity (bottom) were recorded every 1 min. The means of 60 measurements are depicted. Blue: 2014, orange: 2015.

Berlin is situated in the North-East of Germany in the Warsaw-Berlin valley between the Barnim and the Teltow plateaus. The climate is widely continental with cold winters, hot summers and short spring periods. Frequently, like in 2014 and 2015, March is already snow-free with day- and night-time temperatures above −4 °C (Fig. 1 and Suppl. 1). From day 6 of the experiment onwards, the mean temperatures increased in both years. As it is typical for the spring season in Berlin, they dropped again to values below 5 °C, mid of March, end of March, in the first half of April and beginning of May in 2015 and mid of April and in early May in 2014 (Suppl. 1).

The global radiation was 400–580 W m^−2^ on mid-day on the first days of the March experiments (Fig. 1 Middle), which corresponds to a maximum quantum flux of approximately 10 times the light intensity the plants were acclimated to in the growth chamber. In 2014, the maximum and mean radiations (1452 W m^−2^ and 209 W m^−2^) were higher than in 2015 (1385 W m^−2^ and 193 W m^−2^).

The air humidity was around 95% during the nights and between 50 and 65% during the days in the first week of March 2014 (Fig. 1 Bottom). The vegetation period was in general a bit dryer in 2015 than in 2014. The air humidity differed strongest between the two years in June and July, when the seeds matured (Suppl. 1).

### Photosynthetic performance in the field

Temperature and light variation influence photosynthetic electron transport and carbon assimilation^18^,^30^. The maximum and effective quantum yields of photosystem II (F_V_/F_M_ and Φ_PS-II_) are frequently analyzed indicators for photosystem-II (PS-II) damage and regulation^31^,^32^. They can be determined non-destructively by chlorophyll-a fluorescence^32^. As reported recently under exactly the same pre-cultivation conditions, F_V_/F_M_ is 0.83 in Arabidopsis plants directly after 2 weeks at 4 °C due to acclimation^10^. It stays high in the plants, after they are transferred to optimal growth conditions with temperatures around 20 °C^10^. In the figures, the data were arranged according to the LT_50_ values of the accessions for freezing tolerance after 2 weeks a 4 °C (as determined by electrolyte leakage from pretreated leaves at sub-zero temperatures^8^,^28^) (Table 1).

#### March

**Figure 2:**
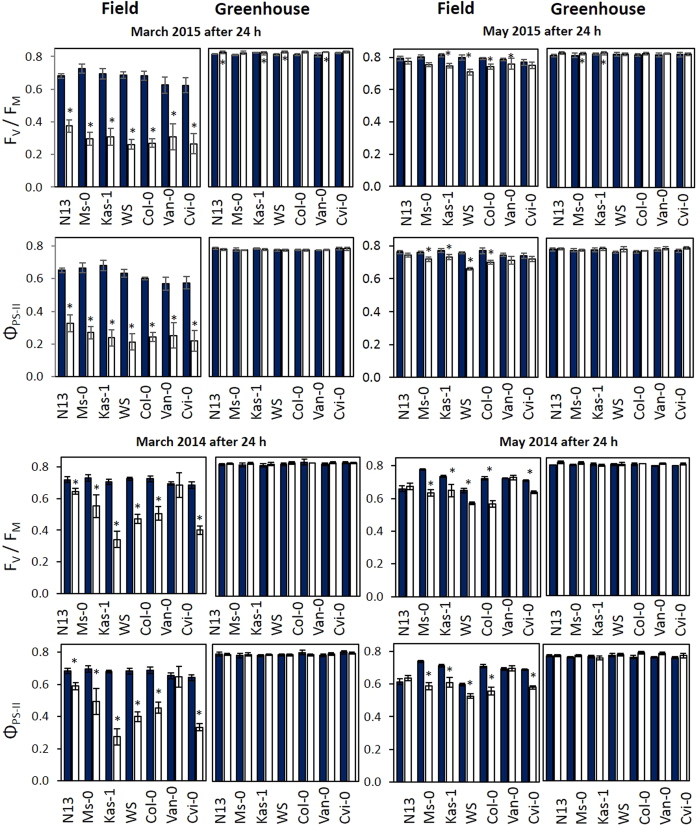
Maximum quantum yields of photosystem II (F_V_/F_M_) and effective quantum yields of photosystem II (Φ_PS-II_) as obtained in cold-pretreated (dark-blue) and naïve plants (white) of the seven accessions by chlorophyll-a fluorescence on the first days of the 2015 (top) and 2014 (bottom) experiments in March (left) and May (right) in the field and in the greenhouse. The asterisks mark statistical significance between cold-treated and naïve plants (ANOVA p < 0.05; n = 6–7).

On the first day after the transfer of cold-pretreated and naïve plants to the field in early March, F_V_/F_M_ and Φ_PS-II_ dropped in all naïve plants (Fig. 2 left). F_V_/F_M_ and Φ_PS-II_ were significantly higher in the cold-pretreated plants. The two accessions with the weakest cold acclimation potential, Van-0 and Cvi-0 had the lowest F_V_/F_M_ and Φ_PS-II_ values. Over all accessions, Φ_PS-II_ correlated with F_V_/F_M_ (r_P_ = 0.996) demonstrating that inactivation of photosystem II dominated over regulation of photosynthetic electron transport.

**Figure 3:**
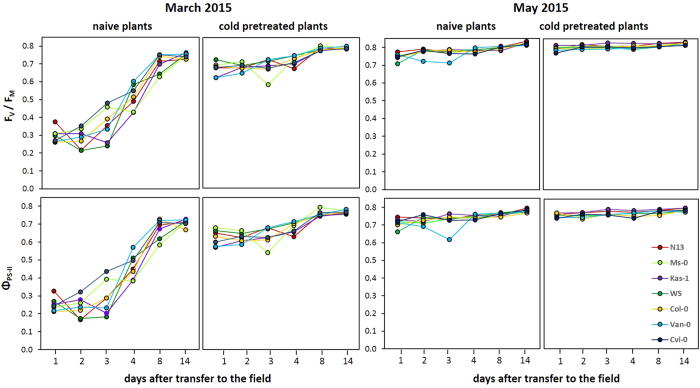
Regulation of the maximum quantum yields of photosystem II (F_V_/F_M_) (top) and effective quantum yields of photosystem II (Φ_PS-II_) (bottom) in the first 14 days of the March (left) and May (right) experiment in cold-pretreated and naïve plants of the seven tested accessions in the field.

Within the first week in the field, F_V_/F_M_ increased in the cold-pretreated plants to levels close to those observed in the greenhouse (Fig. 3 left). In previously naïve plants, except Cvi-0, recovery of the PS-II quantum yield efficiency was delayed (Fig. 3 left). This reaction was slowest in the strongest cold acclimating accessions N13, Ms-0 and Kas-1. In these three accessions, F_V_/F_M_ and Φ_PS-II_ decreased only on day 2 or day 3 in the field. Afterwards PS-II activity recovered, like in the other plants.

In 2014 (Fig. 2 left), F_V_/F_M_ was less decreased in the not cold-pretreated plants than in 2015. The light intensities and humidity were similar in both years beginning of March, but the outdoor daytime temperatures were higher in 2014 on the first four days of the experiment (Fig. 1), indicating that the PS-II activity decrease mainly correlated with temperature sensitivity.

**Figure 4:**
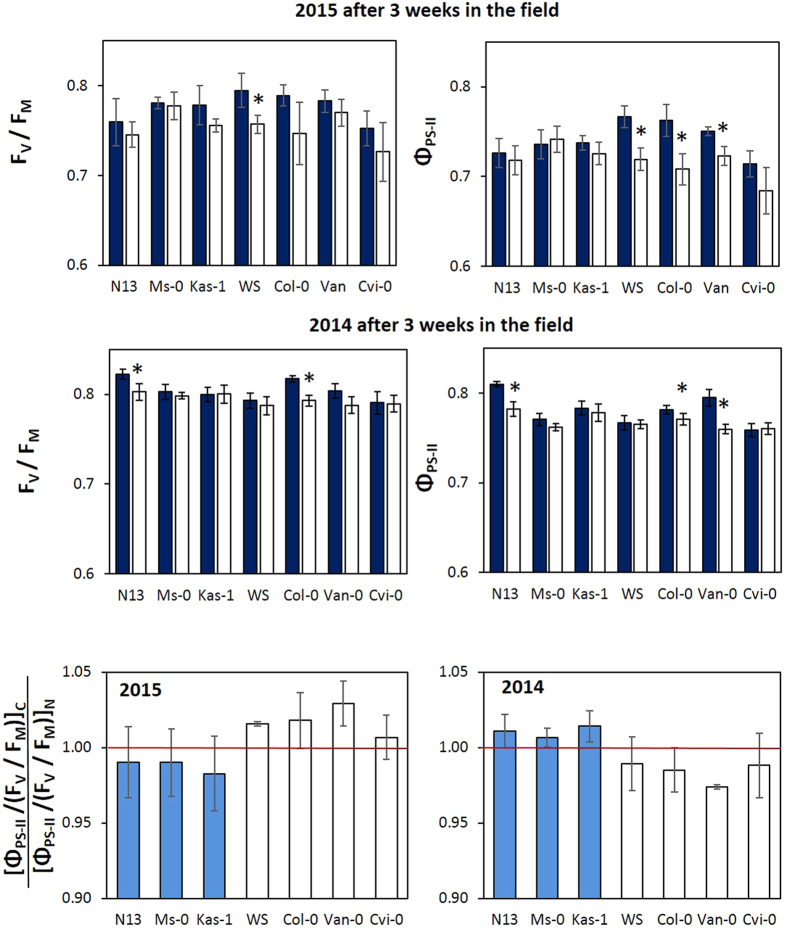
Top and mid: Regulation of the maximum quantum yield of photosystem II (F_V_/F_M_) (left) and effective quantum yield of photosystem II (Φ_PS-II_) (right) on day 21 of the March experiment in cold-pretreated (dark-blue) and naïve plants (white) of the seven tested accessions in the field in 2015 (top) and 2014 (bottom). The asterisks mark statistical significance between cold-treated and naïve plants (ANOVA, p < 0.05; n = 6–7). Bottom: The relative effect of the cold pretreatment as determined by comparison of the relative differences between the ratios of Φ_PS-II_ and F_V_/F_M_ in cold-pretreated (C) and naïve plants (N) in the seven accessions on day 21 of the 2015 (right) and 2014 (left) experiment. The three accessions, N13, Ms-0 and Kas-1, which can acquire a higher cold acclimation potential, are labeled in blue. The red line marks 1.00 (=“no regulatory effect”).

On day 18 and 20 of the March experiment, the temperatures decreased slightly below 0 °C (−2.4 and −1.8 °C) in 2015, but were 3.8–11 °C in 2014 (Fig. 1). On day 21, all accessions, except Ms-0, showed at least slightly higher maximum quantum yields of PS-II (F_V_/F_M_) in the cold-pretreated plants in 2015 (Fig. 4 Top). In the accessions with intermediate ability to acclimate to cold^28^,^8^, namely WS, Col-0 and Van-0, Φ_PS-II_ was higher, if the plants were cold-treated before they were transferred to the field (Fig. 4 Top). The cold pretreatment stronger affected Φ_PS-II_ than F_V_/F_M_, demonstrating that it mainly supported light-dependent activity regulation of PS-II. In the 2014 plant sets, various cold-pretreated plants showed higher F_V_/F_M_ and higher Φ_PS-II_ values still after three weeks (Fig. 4 Top).

For comparison of the cold-priming effect between the accessions, first the Φ_PS-II_ values were normalized on the F_V_/F_M_ values. Afterwards, the results for cold-pretreated plants (C) were divided by the results for naïve plants (N) ([Φ_PS-II_/(F_V_/F_M_)]_C_/[Φ_PS-II_/(F_V_/F_M_)]_N_) (Fig. 4 Bottom). In 2015, the plants faced cold on day 20 of the March experiment after a warmer period (Fig. 1). On the next day, [Φ_PS-II_/(F_V_/F_M_)]_C_/[Φ_PS-II_/(F_V_/F_M_)]_N_ was lower in the three accessions with higher cold acclimation potential than in the other accessions. In 2014, the temperatures were more stable and higher end of March. The [Φ_PS-II_/(F_V_/F_M_)]_C_/[Φ_PS-II_/(F_V_/F_M_)]_N_ effect of 2015 was almost perfectly inverted in 2014 (r_P_ = −0.980) (Fig. 4 Bottom), highlighting the reciprocity of costs and benefits of the cold pretreatment relative to the necessity of cold acclimation in the environment.

#### May

In May, despite approximately more than 15-fold higher radiation than in the growth chamber, F_V_/F_M_ and Φ_PS-II_ were only slightly decreased 24 h after transfer of the plants into the field (Fig. 2 right), but the F_V_/F_M_ and Φ_PS-II_ values were significantly (ANOVA, p < 0.01) lower in naïve plants of all accessions, except in N13, Van-0 and Cvi-0, which have highest and lowest cold-acclimation potential (Table 1). In the greenhouse, like in March, at least slightly higher F_V_/F_M_ were observed in naïve plants of all accessions and higher Φ_PS-II_ values in the accessions WS, Col-0, Van-0 and Cvi-0.

The F_V_/F_M_ and Φ_PS-II_ values also quickly increased in May in the field (Fig. 3 right). On day 2 and day 3 in the 2015 experiment, when the daytime temperatures had reached 20 °C (Fig. 1), the quantum yields of PS-II transiently decreased in not cold-pretreated Kas-1 (Fig. 3 right). Similar, but slighter transient decreases were observed for the other two accessions with high cold acclimation potential, N13 and Ms-0, on day 3 of the experiment.

### Effects of cold pretreatment on the leaf areas

**Figure 5:**
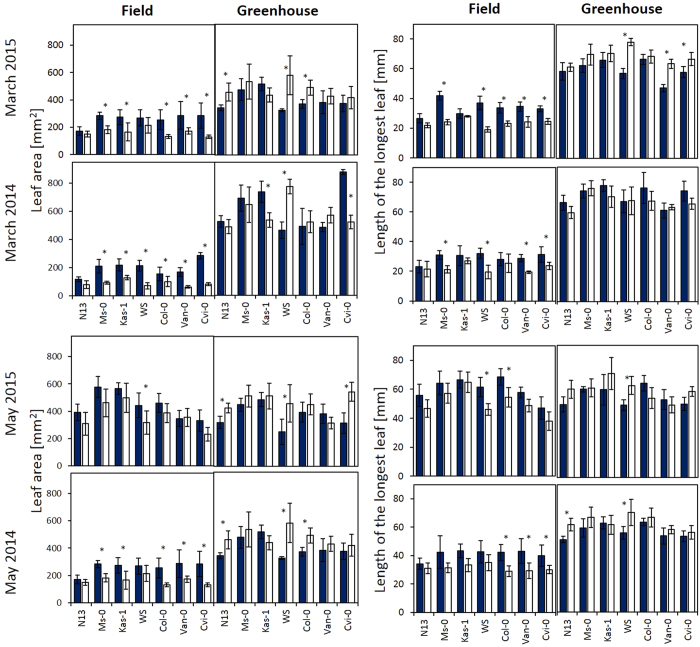
Leaf area in mm^2^ (left) and length of the longest leaf (right) as determined by digital analysis of the largest leaf in cold-pretreated (dark-blue) and naïve plants (white) of seven Arabidopsis accessions 3 weeks after the plants were transferred to the field or into the greenhouse in March and May 2015 and 2014. The asterisks mark statistical significance between cold-treated and naïve plants (ANOVA p < 0.05; n = 5–7).

Leaf area expansion is only partly genetically fixed^33^ and modulated e.g. by the extent of photosynthetic carbon assimilation, which also impacts on the reproductive fitness^34^,^35^. In Arabidopsis, the leaves expand strongest between the 8-leaves-stage (stage 1.08^36^) and bolting (stage 5^36^). Here, we compared the areas of the largest leaves in the rosettes three weeks after transferring the plants to the field and to the greenhouse (Fig. 5 left). These leaves were formed during the pre-cultivation period, but were not fully developed at the time-point of the transfer. By average, the leaf areas of cold-pretreated plants expanded 2.91-fold in the field in the March plant set and 6.04-fold in the greenhouse. In plants transferred to the field in March, the leaf blades of naïve plants, except N13, were at least 50% smaller than in the greenhouse.

In May, the differences between greenhouse and field grown plants were less pronounced (Fig. 5 left). The leaves expanded 6–7-fold within the first three weeks in both growth regimes in 2015. In 2014, when the temperatures were lower than in 2015 in the first days of the May experiment (Fig. 1), leaf expansion was as restricted as in the March transferred plants. However, in general, cold-primed plants had at least slightly larger leaf areas in the field and smaller ones in the greenhouse (Fig. 5 left).

**Figure 6:**
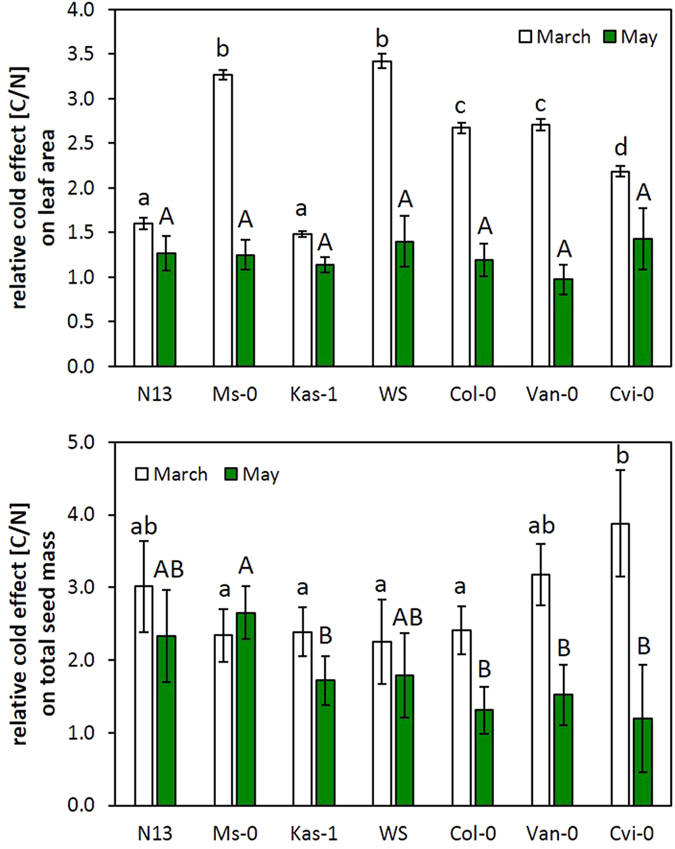
The relative effect of cold pretreatment on the leaf area of the largest leaf 3 weeks after transferring cold-pretreated and naïve plants to the field in March and May (top) and the relative effect of cold pretreatment on the total seed mass per plant in March and May (bottom). The effects were calculated by dividing the values for cold-pretreated plants by the values for naïve plants. Statistically significant differences (ANOVA p < 0.05) between cold-pretreated and naïve plants were labeled with different letters and fonts.

The quotient of the value in cold treated plants and the value in naïve plants [C-value/N-value] shows the priming effect. It was similar in all field-cultivated accessions in the May experiment (Fig. 6 top). In the March experiment, stronger priming effects were observed in WS, Ms-0, Col-0, Van-0 and Cvi-0 than in N13 and Kas-1, which are the accessions with the highest and third highest cold acclimation potential (Table 1). In N13 and Kas-1 only slightly higher priming effects on the leaf blade size were observed showing that strong cold acclimation counteracts the priming effect.

### Effects of cold pretreatment on the leaf and petiole lengths

The length of the longest leaf was determined as an additional morphogenetic parameter summarizing light, metabolite and hormonal regulation^37^ (Fig. 5 right). In the field, the leaves of cold-pretreated Ms-0, WS, Van-0 and Cvi-0 were longer than the leaves of naïve plants in the March experiments. In the greenhouse, no effect was observed in 2014 and longer leaves for WS, Van-0 and Cvi-0 in 2015. The petioles were also slightly longer in cold-treated plants in the field, but the effect was not significant due to high variation between individual leaves (data not shown).

In the May data set, also a trend towards longer leaves was observed after cold pretreatment, but the data were also not significant due to higher variability. In the greenhouse, N13 and WS showed trends to longer leaves in naïve plants.

### Consequences of cold pretreatment on bolting

**Figure 7:**
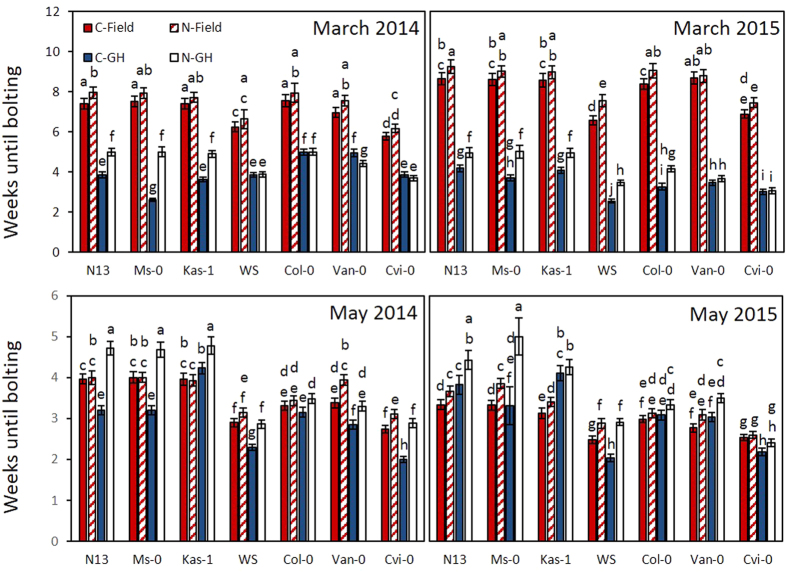
Comparison of bolting times in the field (red) and in the greenhouse (blue/white) started with cold-pretreated (C) and naïve (N) plants of seven accessions in March and May. The data depict means from 16–35 plants per accession and treatment. The data were analyzed for each start-point by ANOVA (Tukey Post-hoc test; p < 0.05) for significance of the differences. Significantly different data are labelled with different letter.

In regions with cold- and/or drought-restricted vegetation periods, the speed of bolting induction can be a critical factor for plant fitness^38^,^39^. Bolting visibly marks the transition from vegetative growth to regenerative growth. It affects leaf expansion, root growth, nutrient uptake and the seed number^40^,^41^. As a measure for bolting induction, we determined the time until the plants had formed at least 1 cm long inflorescences (Fig. 7).

In the March experiments, cold-pretreated plants bolted 3–4 weeks earlier in the greenhouse than in the field. Cold-pretreatment at least slightly accelerated bolting in the field. In the greenhouse, it promoted bolting in the accessions with higher cold acclimation potential (N13, Ms-0 and Kas-1 in both years and in 2015 also in WS and Col-0) (Fig. 7), while Van-0 and Can-0 did not show a priming response on bolting.

In the May experiment (Fig. 7), cold-pretreated Ms-0 and N13 bolted faster than naïve plants in the greenhouse. In the field, these accessions did not show significant differences in 2014. On the contrary, Van-0 and Cvi-0, which have a low cold-acclimation potential (Table 1), bolted earlier if they were cold-pretreated. In 2015, when the temperatures and the humidity were lower than in 2014 (Suppl. 1), N13, Ms-0, Kas-1 and WS bolted earlier in the field if they were cold-primed (Fig. 7 bottom).

### Consequences of cold pretreatment on plant fitness

*Arabidopsis thaliana* is a typical *r*-selected organism^42^: It generates high numbers of small seeds to improve its fitness in habitats with variable environmental conditions. Alternatively, plants can improve their fitness by producing larger and/or heavier seeds, which support the embryo and the seedling with more energy resources^43^. Natural variation of seed size and weight in Arabidopsis is well-known in literature^43^,^44^. The total seed mass is negatively correlated with the number of seeds produced and positively correlated with seedling survival^45^. Here, we quantified the seed mass of individual seeds and, as a mixed parameter for fecundity and quality, the total seed number.

**Figure 8:**
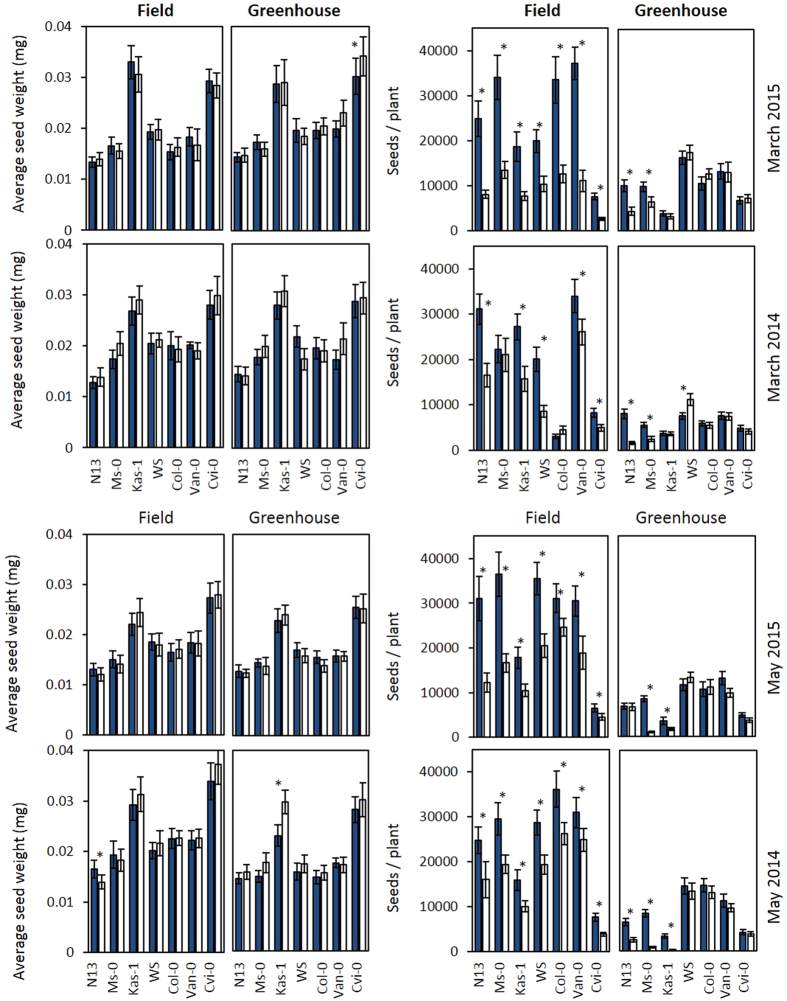
Average weight of individual seeds (left) and number of seeds per plant (right) in the field and the greenhouse experiment in March and May 2015 and 2014. The asterisks marks statistical significance between cold-pretreated (dark-blue) and naïve plants (white) (ANOVA p < 0.05; n = 5–7).

Kas-1 and Cvi-0, which originate from higher altitudes, formed generally slightly heavier seeds, but the cold pretreatment did not affect the individual seed weight in any accession (Fig. 8). However, cold-primed plants of all accessions formed more seeds in the field in the March and in the May experiments. In the greenhouse, only the strongest cold-adapted accessions N13, Ms-0 and Kas-1 (Table 1) produced more seeds (Fig. 8).

To differentiate pretreatment specific and adaptation dependent acclimation effects, the overall fitness effects were compared by normalizing the total seed weight in cold-pretreated plants on the total seed weight in naïve plants (seed weight of C-plants/ seed weight of N-plants) (Fig. 6 bottom). In the March planted series, the two accessions with the lowest cold acclimation potential, Van-0 and Cvi-0, showed the strongest cold effect (Fig. 6 bottom). The strongest absolute response was observed in N13, which was the accession with the highest cold acclimation potential in this study. This result excludes a general correlation of the fitness effects with the cold acclimation potential. On the contrary, the effects of the cold pretreatment on the total seed mass almost gradually decreased from the accessions with high cold acclimation potentials to those with low ones in the May experiment (Fig. 6 bottom).

### Correlation between seed mass and PS-II activity

**Figure 9:**
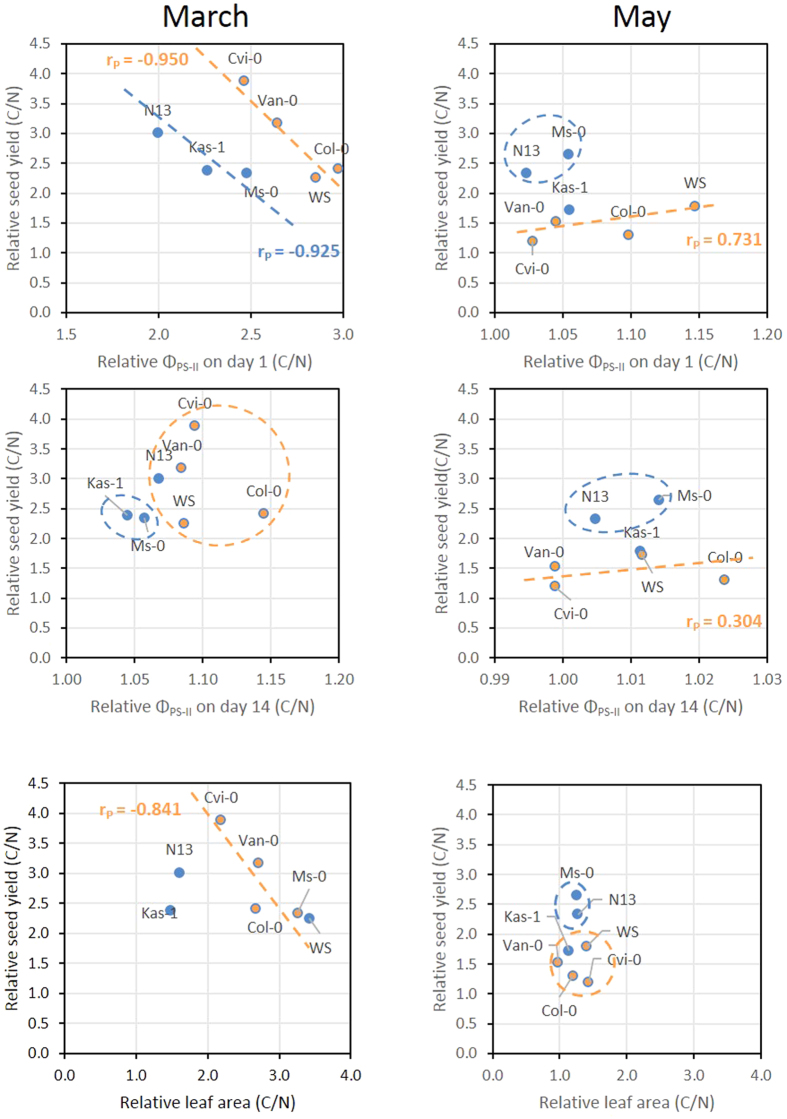
Top and mid: Cluster and correlation analysis of the effect of cold pretreatment on the total seed weight per plant in seven *Arabidopsis thaliana* accessions relative to the total seed weight in naïve plants with the effect of cold pretreatment on the effective quantum yield of photosystem II (Φ_PS-II_) on day 1 (top) and day 14 (mid) in March (left) and May 2015 (right). Clusters identified by squared Euclidean distance analysis and linear correlations are depicted by circles and lines, respectively. The Pearson correlation coefficient (r_P_) is provided as a measure for the intensity of the linear correlations. The three accessions with high cold acclimation potential are depicted in blue, the others in orange. Bottom: Cluster and correlation analysis of the effect of cold pretreatment on the total seed weight per plant in seven accessions relative to the total seed weight in naïve plants with the effect of cold pretreatment on the relative leaf area in March (left) and May 2015 (right). Correlations are depicted by lines, clusters by circles.

To test whether the priming effect on the reproductive fitness (Fig. 8) depends on PS-II regulation, the C-values of both parameters were normalized on the N-values [C-value/N-value]. The 2015 data, which show stronger effect variation than the 2014 data (Figs 2–5), were analyzed for cluster phenomenons and linear correlations. Squared Euclidean distance analysis of the data pairs for seed yield and Φ_PS-II_ on day 1 and day 14 separated N13 and Ms-0 from the accessions with lower cold acclimation potential in the May data set (Fig. 9). The third strongly cold acclimating accession, Kas-1, was placed closest to these clusters (Fig. 9). In the four accessions with intermediate or weak cold acclimation potential, namely WS, Col-0, Van-0 and Cvi-0, the seed yield linearly correlated with the Φ_PS-II_ on day 1 (r_P_ = 0.731) and vaguely with Φ_PS-II_ on day 14 (r_P_ = 0.304) (Fig. 9).

In the March data sets squared Euclidean distance analysis did not support separation of stronger and weaker cold acclimatible accessions in distinct clusters. Correlation analysis for the stronger cold acclimating accessions N13, Ms-0 and Kas-1 and for the four other accessions showed high negative linear correlation (r_P_ = −0.950 for Cvi-0, Van-0, Col-0 and WS and r_P_ = −0.925 for N13, Kas-1 and Ms-0) between the priming effect on the seed yields and the priming effect on Φ_PS-II_ on the first day in the field (Fig. 9). For N13, Ms-0 and Kas-1 a similar difference in Φ_PS-II_ between cold-pretreated and naïve plants resulted in lower seed yields. The correlation lines of the two groups of accession was almost parallel shifted and separated the accessions into two regulatory units.

The leaf area after 3 weeks in the field almost perfectly correlated (r_P_ = 0.999) with the total seed mass of naïve plants in the March experiments. The correlation was lost in the cold-adapted accessions (N13, Ms-0, Kas-1) and almost fully inverted (r_P_ = −0.841) in the other accessions for the priming effects [C-value/N-value] on the leaf areas and the seed yields (Fig. 9 bottom left). In the May experiment, variation of the leaf areas was very low (Fig. 9 Bottom right). Ms-0 and N13 clustered separately from the other accessions due to slightly higher seed yields (Fig. 9 Bottom right).

## Discussion

### Costs and benefits of cold pretreatment under stable environmental conditions

According to the allocation cost theories, attribution of energy and metabolites into defense and acclimation limit growth and reproduction^46^,^47^,^48^. The costs of the cold pretreatment can be observed if acclimated plants are transferred to non-stress conditions. Here, cold pretreatment resulted in smaller leaf blades and (except in March 2014) in shorter leaves in the greenhouse in various accessions (Fig. 5) indicating resource restriction^49^. The seed numbers or seed weights were not decreased (Fig. 8) demonstrating that the cold pretreatment impacted not on the reproductive fitness.

The accessions N13, Ms-0 and Kas-1 hardly produced seeds in the greenhouse without cold pretreatment (Fig. 8; greenhouse data). These accessions originate from areas with shorter vegetation periods and long cold winters (Table 1) and have stronger vernalization dependent alleles of *FLC* (*FLOWERING LOCUS C*) and *FRI* (*FRIGIDA*)^5^,^50^. *FLC* and *FRI* control meristem transition^51^ and support cold acclimation^5^,^38^,^52^. The release of osmolytes, e.g. trehalose^53^,^54^, also supports meristem transition and induces bolting. N13, Ms-0 and Kas-1 accumulate more osmolytes in the cold than the other accessions^8^. The fast release of higher amounts of osmolytes upon early deacclimation^8^ provides a stronger metabolite flux in the cold-pretreated plants in the greenhouse. In the field, the accessions also flowered efficiently without cold-pretreatment. N-plants of the accessions N13, Ms-0 and Kas-1 formed similar or even more seeds in the field than cold-pretreated plants in the greenhouse did (Fig. 8).

### Effect of cold pretreatment on bolting in the field

In May, the plants were exposed to long-day conditions in the field similar to the greenhouse illumination regime, but the temperatures were lower, especially during the nights (Fig. 1). While photoperiod sensing should have supported meristem transition^55^,^56^,^57^, bolting was only slightly (May 2015) or not promoted (May 2014) in the accessions with high cold acclimation potential (N13, Ms-0 and Kas-1). Low night temperatures in combination with diurnal temperature variations activate cold acclimation stronger than continuous cold stress does^7^. Decreased conveyance of osmolytes into cold acclimation can explain the delay in bolting based on the allocation cost theories^46^,^47^,^48^.

As shown in the Arabidopsis mutant *gigantea* and by application of ascorbate, stronger antioxidant protection delays flowering^58^,^59^,^60^. At low temperatures, ascorbate accumulates and the genes for ascorbate and glutathione regenerating enzymes are expressed stronger^10^,^24^,^61^. Consistent with the bolting effect (Fig. 7), the more cold adapted accessions decrease the ascorbate pool size more slowly after cold acclimation than the accessions with lower cold acclimation potential^24^.

### Benefits of cold adaptation on the reproductive fitness

Cold-pretreated plants produced more seeds than naïve ones in the field in the May experiment (Fig. 8). The effect was widely independent from changes in the leaf area and the photosynthetic activity (Figs 2 and 5). The total seed mass in cold-pretreated plants relative to that in naïve plants (C/N-ratio) gradually decreased from the accessions with higher cold acclimation potentials to the accessions with the lower ones (Fig. 6 bottom). The best cold adapted accessions N13, Ms-0 and Kas-1 activate the CBF-regulon stronger in the cold^8^. Either directly or via its upstream regulator ICE1, cold impacts on the circadian clock, FLC-regulation and hormone, redox and phytochrome signaling^62^,^63^,^64^ and controls stress protection, which supports the reproductive fitness^65^,^66^. The trend observed here is consistent with a large body of literature demonstrating that cold acclimation supports the reproductive fitness in the field.

### Costs of a high cold acclimation potential

Harsher weather conditions (Fig. 1) caused stronger effects in the field in March than in May (Figs 2–6). As frequently reported in literature (for review: refs 11 and 18), cold-pretreated plants benefit in the cold from previous cold acclimation by better PS-II protection (Fig. 2 left). In our study, F_V_/F_M_ decreased on the first and/or second day after transfer of the plants to the field in naïve N13, Ms-0 and Kas-1 (Fig. 2 left) demonstrating weaker stabilization of PS-II activity against natural environmental conditions with e.g. light and temperature fluctuations. The other accessions did not show the effect. Lower PS-II activity in the best cold adapted accessions demonstrated costs of cold acclimation. The difference in the dynamic of PS-II activity recovery (Fig. 3 left) is not linked to regulation of photosynthetic electron transport as cold acclimation results in more efficient Q_A_ oxidation^67^. Weaker expression of plastid antioxidant enzymes in the cold^24^ increases the risk for photodamage^68^,^69^ and inhibits PS-II^69^,^70^,^71^. Here, PS-II was inhibited stronger in naïve plants, if the plants were exposed to the field conditions (Fig. 2 left). Recovery often includes proteolytic digestion, protein *de novo* synthesis and photosystem re-assembly^72^. These processes take time and are temperature sensitive^73^. The weaker chloroplast antioxidant protection in accessions with a higher cold acclimation potential^24^ delays the recovery of PS-II activity and can explain the slower response in N13, Ms-0 and Kas-1 (Fig. 3 left).

### Differentiating cold acclimation and cold priming responses

The benefit of the cold-pretreatment on the seed yield was higher in the best and also in the worst cold-adapting accessions, after the plants were transferred to the field in March (Fig. 6 bottom). The two-directional effect indicates an overlay of two parallel acting seed yield supporting mechanisms. As shown recently under growth chamber conditions in the Arabidopsis accession Col-0^10^, protection by cold acclimation is accompanied by independently regulated priming effects, such as mitigating chloroplast ROS signaling and strengthening pleiotropic stress responses. In contrast to acclimation, priming is not coupled to the persistence of a stressful situation. Priming is defined as a mechanism transmitting information on a previous stress over a stress-free period^74^. Comparison of 24 h and 14 day long cold pretreated plants demonstrated that the response to cold priming decreases if the first stress stimulus is long enough to activate cold acclimation^10^. We concluded that cold priming is antagonized by cold acclimation^10^. Cold-pretreated accessions showed better PS-II activity stabilization than naïve plants after 3 weeks (Fig. 4) demonstrating positive cold priming effects in the field (Fig. 4). PS-II activity was indistinguishable from that in naïve plants already 1 week after the transfer (Fig. 3 left). One day before the chlorophyll-a fluorescence analysis (day 20), the plants faced a triggering stimulus after a cold-free period (Fig. 1; Suppl. 1). Consistent with the definition of priming^74^ and with the previous hypothesis that priming acts independently from cold acclimation and is of importance in nature in spring^10^, the cold pretreatment resulted in higher Φ_PS-II_ in the intermediate cold-adapted accessions WS, Col-0 and Van-0 than in the most cold-adapted accessions N13, Ms-0 and Kas-1 (Fig. 4). The priming memory depends on post-stress regulation of the chloroplast antioxidant system, which stabilizes PS-II activity^75^,^76^. It antagonizes chloroplast-to-nucleus ROS signaling, while chloroplast-independent ROS signaling is unaffected^10^. A triggering stress induces pleiotropic stress protection stronger in primed plants^10^. Here, the priming effect on the total seed mass correlated negatively with the priming effect on Φ_PS-II_ at day 1 after the transfer into the field (Fig. 9; March) demonstrating that the protective effect is independent from the direct effect of cold acclimation, but controlled by priming-induced regulation.

## Conclusion

Cold pretreatment supported the reproductive fitness in all tested accessions in the field, regardless whether the plants were transferred in March or in May. In March, the effect on the total seed mass was linked to stabilization of Φ_PS-II_. In the plants transferred in May, it negatively correlated with the cold acclimation potential independent of effects on PS-II activity, which excludes metabolite allocation and metabolite signaling effects. The March transferred plants experienced cold in the field. In this plant group, the cold-pretreatment supported the seed yield the most in the two accessions with the lowest cold acclimation potential, Van-0 and Cvi-0. Higher Φ_PS-II_ after 3 weeks in the field furthermore demonstrated that accessions with weaker cold acclimation potential benefited more from cold acclimation than accessions with high, adapted cold tolerance.

## Methods

### Plant material and growth conditions

The *Arabidopsis thaliana* accessions Col-0 (INRA Accession ID 186AV), Cvi-0 (166AV), Kas-1 (434AV), Ms-0 (93AV), N13 (266AV), Van-0 (161AV) and WS (84 AV) were propagated in the greenhouse. The seeds of parallelly grown plant sets of 60 (2014) and 160 plants per accession were stratified in darkness for three days at 4 °C on Arabidopsis soil (70 volumes “Topferde” (Einheitserde, Germany), 70 volumes “Pikiererde” (Einheitserde, Germany), 25 volumes Perligran Classic (Knauf, Germany) supplemented with 0.5 g l^−1^ dolomite lime (Deutsche Raiffeisen-Warenzentrale, Germany)) and watered with 0.5 g l^−1^ Axoris Insekten-frei (COMPO, Germany) prior to transfer to a climate-controlled chamber with a day/night rhythm of 10 h at 20 ± 2 °C and 120 μmol photons m^−2^ s^−1^ light (L36W/840 Lumilux Cool White fluorescent tubes) and 14 h at 18 ± 2 °C in darkness at 60 ± 5% relative humidity. At an age of 28 days, half of the plants were transferred to 4 °C and cultivated for 14 days in the 10 h light/14 h dark rhythm with the same illumination, aeration settings and humidity as in the growth chamber. Up to this point of the experiment, the cultivation conditions were identical to those used in the LTC (long term cold) data set in the previous study^10^.

**Figure 10:**
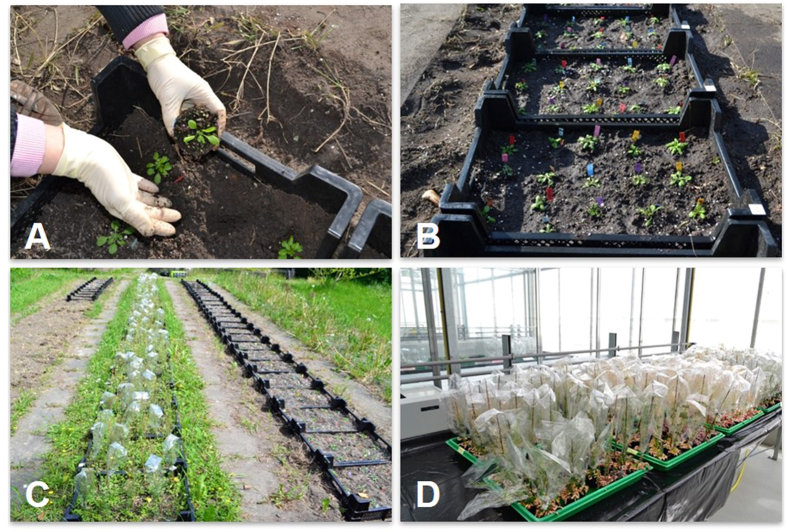
Experiments in March and May. (**A** + **B**) Field-experiment: Cold-pretreated and naïve plants were planted in an open basket system in randomized patterns. (**C**) Field experiment: At the time the May experiment was started in the outer lanes, most plants of the March-experiment started to set seeds, which were collected in cellophane bags. (**D**) Greenhouse experiment: the plants were grown in randomized patterns in trays. Like in the field, the seeds were collected on the plants.

15 (2014) and 35 (2015) cold-pretreated plants (C) and the same numbers of untreated (naïve) plants (N) per accession were transferred to the field (Fig. 10). For optimal soil contact, the pots were removed. The field areas were covered with a natural weed vegetation prior to the experiment. They have not been treated with any fertilizer or herbicide for at least 5 years and were prepared by 20–25 cm deep ploughing one week before the experiments were started. Slug interference was minimized by application of ‘Mesurol Schneckenkorn’ pellets (Bayer, Germany). The same numbers of the cold-treated and naïve plants were transferred to a temperature (20 °C during the 14 h long day period and 18 °C during the night), light intensity (illumination with 55 klux with Philips SON-T AGRO, if the external light intensity was <50 klux during the day phase, and activation of shading, if the external light intensity was >55 klux, which corresponds to 100–120 μmol photons m^−2^ s^−1^ on plant rosette level) and humidity controlled (45% relative humidity) greenhouse. The position of accessions and treatments were randomized between and inside the trays and baskets. The plants were cultivated in the field and in the greenhouse until the end of the seed harvest (Fig. 8). Data from plants, which were damaged during the experiment, were removed from the data set. For the final data analysis, only data obtained with the same plants were used.

### Chlorophyll-a fluorescence analysis

The maximum quantum efficiency of photosystem II (F_V_/F_M_ = (F_M_ − F_0_)/F_M_) and the effective quantum yield of photosystem II (Φ_PSII =_  = (F_M′_ − F_0_)/F_M′_), were determined by chlorophyll-a fluorescence analysis using a MINI-PAM fluorimeter (Walz, Effeltrich, Germany)^32^,^77^ and “Dark leaf clips DLC-8” (Walz, Effeltrich, Germany). The measurement was started with open leaf clips at 10:30 a.m. by determination of Φ_PSII_ in the center of one leaf half of the largest leaf with a 0.8 s long saturating light flash (>3000 μmol photons m^−2^ s^−1^). The leaf clips were placed in 25 sec intervals on the various plants. The measurements were started with determination of Φ_PSII_. 20 min after closing the leaf clips for dark acclimatization, F_V_/F_M_ was determined in the various plants in the same rhythm as determination of Φ_PSII_ on the same leaf and the same position. Afterwards, the leaf clips were removed.

### Plant imaging analysis

Top views of the plants were taken weekly with a digital camera in the field and greenhouse. The pictures were analyzed with the ImageJ software package^78^ for the leaf length and petiole length in mm and the leaf area in mm^2^.

### Determination of seed numbers and seed weight

The inflorescences were bagged in cellophane before the first siliques opened (Fig. 10C and D). The seeds were air-dried in the bags for 4 weeks at room temperature. After cleaning them from attached petals, sepals and silique valves, the seeds were incubated in the dark and at room temperature for 6 more weeks, before the total seed mass per plant (n = 15) and the average individual seed weight were determined by weighting all seeds and three aliquots of 100–200 seeds on a digital balance (ABT320-4B, Kern & Sohn GmbH, Germany). The exact seed number was determined for each aliquot of seeds originating from 6–7 plants used in the chlorophyll-a fluorescence analysis on digital images using the ‘Analyze particles’ function of the software package ImageJ^78^. The parameter ‘Size’ was set to 6–150 and the ‘Circularity’ to 0.7–1.0 for counting. The individual seed weights were calculated by dividing the seed weight of the aliquots by the number of seeds counted in the respective aliquot. The total number of seeds per plant was calculated from the total seed weight and the mean individual seed weight in the same batch.

### Weather data

The air temperature and the relative air humidity were determined 2 m above the ground and the global radiation was obtained at the meteorological station ‘Dahlem’ of the Free University of Berlin (Institut für Meteorologie der Freien Universität Berlin, Germany) 610 m east and 750 m southeast of the site. All parameters were recorded at one-minute intervals from the beginning of March to the time of the last harvest. For the figures, one-hour-mean values were calculated. Additionally, in the field, the quantum flux (quantum flux [μmol quanta m^−2^ s^−1^] = radiation [Wm^−2^]∙λ [nm]∙0.836∙10^−2^) was recorded with a MQ-200 quantum flux meter (Apogee Electronics, Santa Monica CA, U.S.A.) and the air temperature and humidity controlled with a Voltcraft DL-121TH data logger (Voltcraft, Switzerland) several times during the experiment for comparison.

### Statistical analyses

The ANOVA (Turkey Post-hoc test), cluster analysis (squared Euclidean distance; cluster number set to 2–5 clusters) and Pearson correlation analyses were carried out at α < 0.05 using R (version 3.2.2, Vienna, Austria), SPSS 22 and 23 (IBM Armonk U.S.A.) and Excel (Microsoft, Redmond, U.S.A.).

## Additional Information

**How to cite this article**: Cvetkovic, J. *et al*. The effect of cold priming on the fitness of *Arabidopsis thaliana* accessions under natural and controlled conditions. *Sci. Rep.* **7**, 44055; doi: 10.1038/srep44055 (2017).

**Publisher's note:** Springer Nature remains neutral with regard to jurisdictional claims in published maps and institutional affiliations.

## Supplementary Material

Supplementary Information1

## Figures and Tables

**Table 1:** Geographic data of the collection sites for the *Arabidopsis thaliana* accessions used in this study.

Accession	Latitude of collection site	Longitude of collection site	LT_50_ [°C] non-acclimated	LT_50_ [°C] after 2 weeks at 4 °C
N13	N 62.12	E 34.01	−5.52	−11.76
Ms-0	N 55.45	E 37.35	−7.72	−11.87
Kas-1	N 34.00	E 76.00	−4.71	−11.86
Ws-0	N 52.22	E 30.38	−6.02	−10.38
Col-0	N 52.73	E 15.15	−5.34	−9.13
Van-0	N 49.16	W 123.07	−6.03	−8.79
Cvi-0	N 16.00	W 24.00	−4.98	−7.78

The LT_50_ values were taken from refs 8 and 28 and reflect the temperature, at which 50% of electrolyte leakage takes place.
