# Supplementary material for: The effect of cold priming on the fitness of Arabidopsis thaliana accessions under natural and controlled conditions
Source: Sci Rep. 2017 Mar 9;7:44055. doi: 10.1038/srep44055 (PMC5343467; doi:10.1038/srep44055)
Supplement: Supplementary Information1 [file srep44055-s1.pdf]

Supplement 1

„The effect of cold priming on the fitness of *Arabidopsis thaliana* accessions under natural and controlled conditions"

Jelena Cvetkovic, Klaus Müller and Margarete Baier

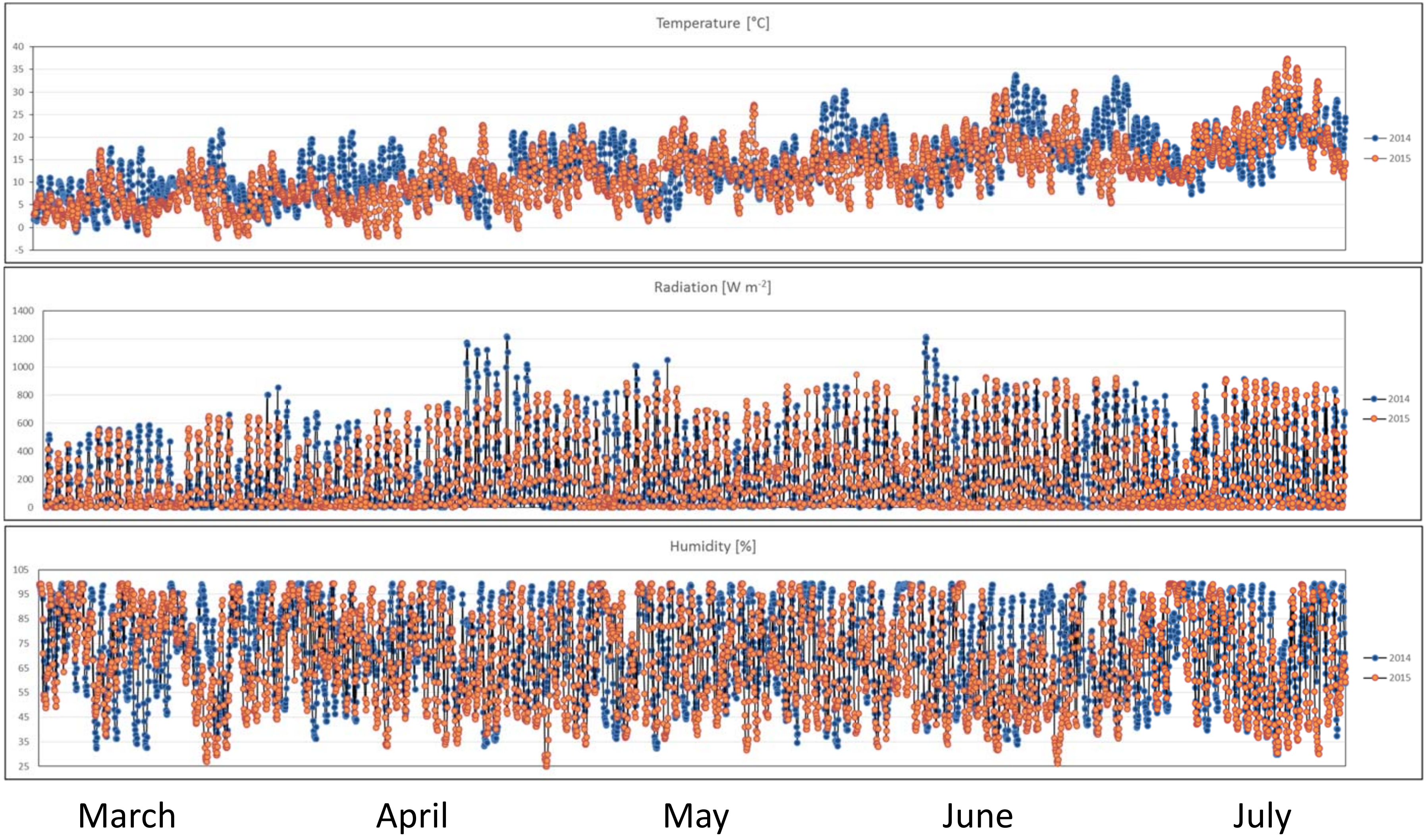

Supplement 1: Weather data for beginning of March until end of July. The mean temperature, global radiation and relative humidity were recorded every 1 min. The means of 60 measurements are depicted. Blue: 2014, orange: 2015.
